# 3-Ethenyl-1-(4-methyl­phenyl­sulfon­yl)-1*H*-indole

**DOI:** 10.1107/S1600536812021526

**Published:** 2012-05-19

**Authors:** Julio Zukerman-Schpector, Glaudeston D. Wulf, Hélio A. Stefani, Stanley N. S. Vasconcelos, Seik Weng Ng, Edward R. T. Tiekink

**Affiliations:** a Departmento de Química, Universidade Federal de São Carlos, CP 676, 13565-905 São Carlos, SP, Brazil; b Departamento de Farmácia, Faculdade de Ciências Farmacêuticas, Universidade de São Paulo, São Paulo, SP, Brazil; c Department of Chemistry, University of Malaya, 50603 Kuala Lumpur, Malaysia; d Chemistry Department, Faculty of Science, King Abdulaziz University, PO Box 80203 Jeddah, Saudi Arabia

## Abstract

Two independent but very similar mol­ecules comprise the asymmetric unit of the title compound, C_17_H_15_NO_2_S. The mol­ecules have L-shapes with the dihedral angles between the fused-ring system (r.m.s. deviations = 0.036 and 0.019 Å, respectively) and the benzene ring being almost the same, *i.e*. 82.98 (12) and 84.46 (13)°, respectively. The terminal ethenyl group is almost coplanar with the ring to which it is connected [C—C—C—C torsion angles = −173.7 (4) and −171.7 (4)°, respectively]. Supra­molecular arrays parallel to (-124) stabilized by C—H⋯O and C—H⋯π inter­actions feature in the crystal packing.

## Related literature

For background to the biological activity of indole­amine 2,3-di­oxy­genase and inhibitors, see: Rohrig *et al.* (2010 ▶); Munn & Mellor (2007 ▶); Muller *et al.* (2005 ▶). For related structures, see: Seshadri *et al.* (2002 ▶); Senthil Kumar *et al.* (2006 ▶); Chakkaravarthi *et al.* (2008 ▶).
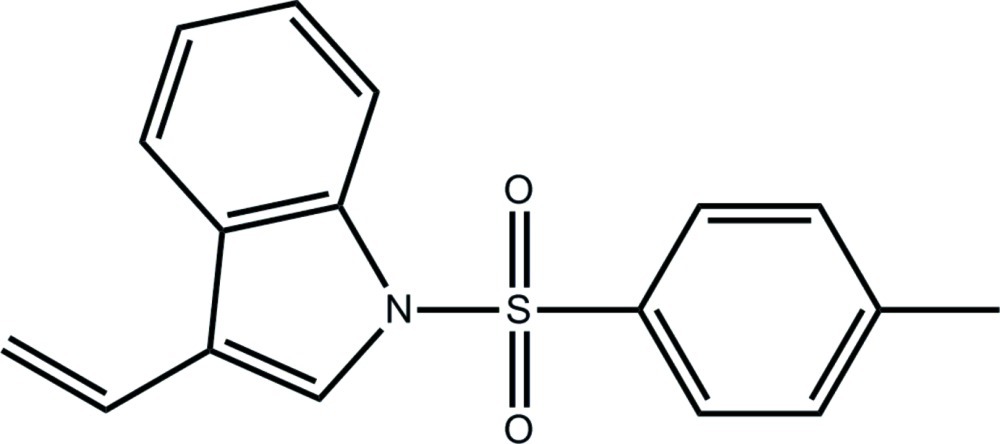


## Experimental

### 

#### Crystal data

C_17_H_15_NO_2_S
*M* *_r_* = 297.37
Triclinic, 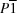

*a* = 9.8809 (4) Å
*b* = 10.0167 (3) Å
*c* = 15.5280 (5) Å
α = 83.687 (3)°
β = 77.864 (3)°
γ = 88.769 (3)°
*V* = 1493.41 (9) Å^3^
*Z* = 4
Cu *K*α radiation
μ = 1.95 mm^−1^
*T* = 100 K
0.35 × 0.30 × 0.25 mm

#### Data collection

Agilent SuperNova (Dual, Cu at zero) diffractometer with an Atlas detector
Absorption correction: multi-scan (*CrysAlis PRO*; Agilent, 2010 ▶) *T*_min_ = 0.548, *T*_max_ = 0.641
11566 measured reflections
6103 independent reflections
5505 reflections with *I* > 2σ(*I*)
*R*_int_ = 0.019

#### Refinement

*R*[*F* ^2^ > 2σ(*F* ^2^)] = 0.069
*wR*(*F* ^2^) = 0.185
*S* = 1.02
6103 reflections
381 parameters
H-atom parameters constrained
Δρ_max_ = 0.94 e Å^−3^
Δρ_min_ = −0.46 e Å^−3^

### 

Data collection: *CrysAlis PRO* (Agilent, 2010 ▶); cell refinement: *CrysAlis PRO*; data reduction: *CrysAlis PRO*; program(s) used to solve structure: *SIR92* (Altomare *et al.*, 1999 ▶); program(s) used to refine structure: *SHELXL97* (Sheldrick, 2008 ▶); molecular graphics: *ORTEP-3* (Farrugia, 1997 ▶), *QMol* (Gans & Shalloway, 2001 ▶), *DIAMOND* (Brandenburg, 2006 ▶) and *MarvinSketch* (ChemAxon, 2009 ▶); software used to prepare material for publication: *publCIF* (Westrip, 2010 ▶).

## Supplementary Material

Crystal structure: contains datablock(s) global, I. DOI: 10.1107/S1600536812021526/hg5227sup1.cif

Structure factors: contains datablock(s) I. DOI: 10.1107/S1600536812021526/hg5227Isup2.hkl

Supplementary material file. DOI: 10.1107/S1600536812021526/hg5227Isup3.cml

Additional supplementary materials:  crystallographic information; 3D view; checkCIF report

## Figures and Tables

**Table 1:** Hydrogen-bond geometry (Å, °) *Cg*1, *Cg*2 and *Cg*3 are the centroids of the C1–C6, N2–C25 and C18–C23 rings, respectively.

*D*—H⋯*A*	*D*—H	H⋯*A*	*D*⋯*A*	*D*—H⋯*A*
C8—H8⋯O2^i^	0.95	2.50	3.433 (4)	166
C20—H20⋯O2^ii^	0.95	2.52	3.373 (5)	149
C25—H25⋯O4^iii^	0.95	2.48	3.406 (4)	166
C30—H30⋯*Cg*1^iv^	0.95	2.77	3.617 (4)	149
C34—H34*C*⋯*Cg*2^v^	0.98	2.95	3.525 (4)	119
C12—H12⋯*Cg*3^v^	0.95	2.87	3.739 (3)	153
C15—H15⋯*Cg*3^v^	0.95	2.86	3.638 (3)	140

Symmetry codes: (i) 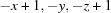; (ii) 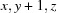; (iii) 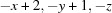; (iv) 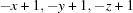; (v) 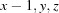.

**Fig. 1.**
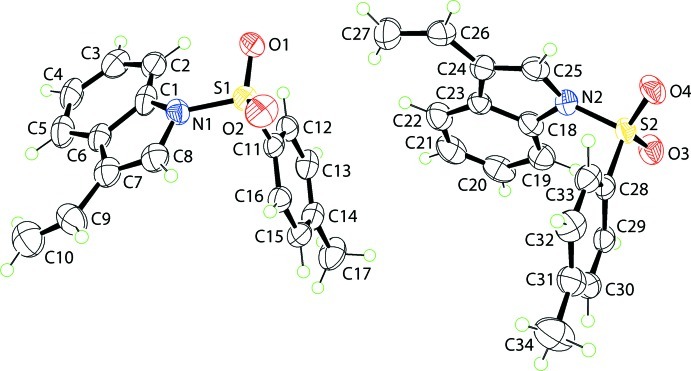
The molecular structures of the two independent molecules in (I) showing atom labelling scheme and displacement ellipsoids at the 50% probability level (arbitrary spheres for the H atoms).

**Fig. 2.**
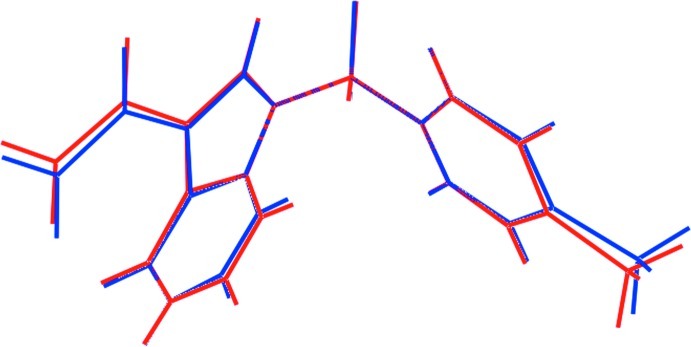
Overlay diagram for the S1- (red) and S2-containing (blue) molecules aligned so that the N1—S1—C11 and N2—S2—C28 atoms were coincident.

**Fig. 3.**
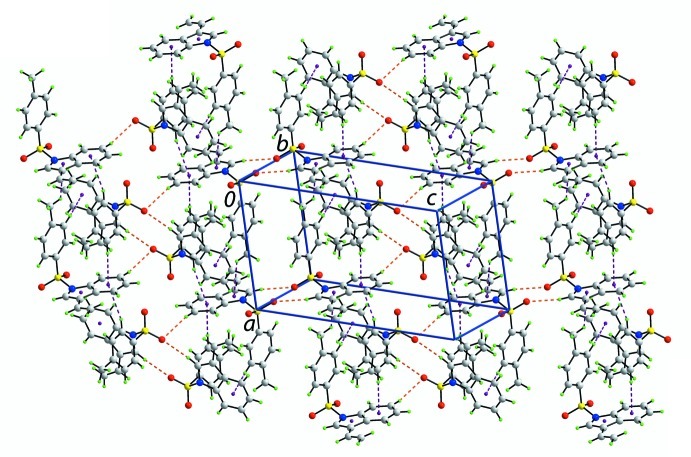
A view of the supramolecular array parallel to (1 2 4) in (I). The C—H···O and C—H···π interactions are shown as orange and purple dashed lines, respectively.

**Fig. 4.**
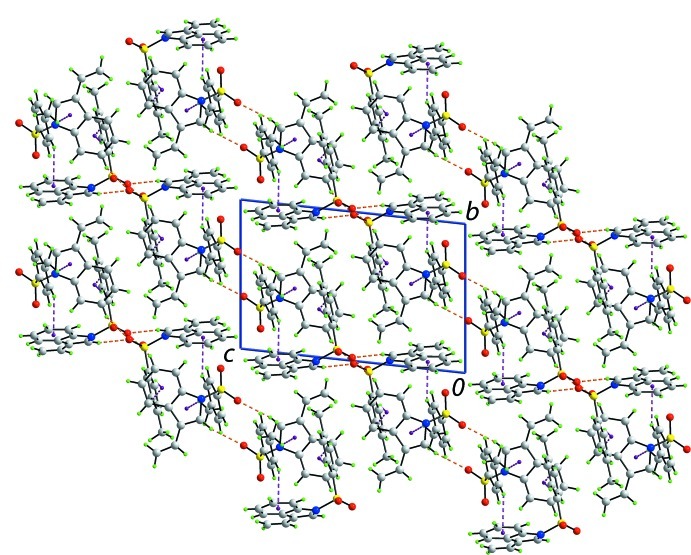
A view in projection down the a axis of the unit-cell contents of (I) highlighting the stacking of supramolecular layers. The C—H···O and C—H···π interactions are shown as orange and purple dashed lines, respectively.

## supplementary crystallographic information

### Comment

Indoleamine 2,3-dioxygenase (IDO) is an enzyme that catalyses the degradation of the essential amino acid tryptophan. Elevated tryptophan catabolism mediated by IDO is associated with a wide variety of human cancers and cataract formation (Rohrig *et al.*, 2010). It has also been shown that inhibition of IDO leads to an arrest in tumour growth (Munn & Mellor, 2007; Muller *et al.*, 2005). As part of our on-going research targeted towards the synthesis of α- and β-hydroxy indols as potential IDO inhibitors, the title compound, (I), was synthesized and its crystal structure determined.

There are two independent molecules in the asymmetric unit of (I), Fig. 1, and as seen from the overlay diagram in Fig. 2, these are almost identical with the r.m.s. deviation being 0.1107 Å. The dihedral angles between the fused ring system (r.m.s. deviations = 0.036 and 0.019 Å for the N1- and N2-containing rings, respectively) and the benzene ring are almost the same, *i.e*. 82.98 (12) and 84.46 (13)°, respectively. The values found in similar structures are of 80.37 (8)° (Chakkaravarthi *et al.*, 2008), 77.41 (5)° (Senthil Kumar *et al.*, 2006) and 66.47 (15) °. (Seshadri *et al.*, 2002). For each molecule, the terminal ethenyl group is almost co-planar to the ring to which it is connected as seen in the values of the C8—C7—C9—C10 and C25—C24—C26—C27 torsion angles of -173.7 (4) and -171.7 (4)°, respectively.

The crystal packing of (I) is sustained by C—H···O and C—H···π interactions, Table 1. These lead to supramolecular arrays parallel to (1 2 4), Fig. 2, which stack with no specific intermolecular interactions between them, Fig. 3.

### Experimental

A solution of methyltriphenylphosphonium iodide (0.34 g, 0.84 mmol, 1.4 eq.) in THF (5 ml) at 273 K was poured into a two-necked round-bottomed flask under a nitrogen atmosphere and then under continuous stirring *n*BuLi (0.36 ml, 0.72 mmol,1.2 eq.) was added drop-wise at 195 K. The mixture was left in a water/ice bath for 20 min, then a solution of 1-tosyl-1-*H*-indol-carbaldehide (0.181 g) in THF (5 ml) was added. After stirring for another 20 min. the solution was warmed to room temperature and water added. The mixture was extracted with Et_2_O, washed with NH_4_Cl and dried under MgSO_4_. The remaining solvent was removed under reduced pressure. Purification through flash chromatography with a solution of hexane and ethyl acetate in a 7:3 ratio give the pure product (yield = 62%). Crystals for X-ray analysis were obtained by slow evaporation from EtOAc held at 293 K; *M*.pt: 374–375 K.

NMR ^1^H (CDCl_3_, 300 MHz, p.p.m.): δ 7.99 (d, J = 8.2 Hz, 1*H*), 7.75 (d, J = 8.4 Hz, 2*H*), 7.72 (d, J = 8.3 Hz, 1*H*), 7.59 (s, 1*H*), 7.34–7.23 (m, 2*H*), 7.17 (d, J = 8,2 Hz, 2*H*), 6.75 (dd, J = 17.8 and 11.3 Hz, 1*H*), 5.78 (d, J = 17.8 Hz, 1*H*), 5.33 (d, J = 11,3 1*H*), 2.29 (s, 3*H*). NMR ^13^C (CDCl_3_, 75 MHz, p.p.m.): δ 145.04, 135.55, 135.19, 129.91 (2 C), 129.04, 127.57, 126.85 (2 C), 124.92, 124.09, 123.53, 121.00, 120.43, 115.35, 113.76, 21.54.

### Refinement

The H atoms were geometrically placed (C—H = 0.95–0.98 Å) and refined as riding with *U_iso_*(H) = 1.2–1.5*U_eq_*(C).

### Crystal data

**Table:**

C_17_H_15_NO_2_S	*Z* = 4
*M**_r_* = 297.37	*F*(000) = 624
Triclinic, *P*1	*D*_x_ = 1.323 Mg m^−^^3^
Hall symbol: -P 1	Cu *K*α radiation, λ = 1.54184 Å
*a* = 9.8809 (4) Å	Cell parameters from 5330 reflections
*b* = 10.0167 (3) Å	θ = 2.9–75.8°
*c* = 15.5280 (5) Å	µ = 1.95 mm^−^^1^
α = 83.687 (3)°	*T* = 100 K
β = 77.864 (3)°	Prism, colourless
γ = 88.769 (3)°	0.35 × 0.30 × 0.25 mm
*V* = 1493.41 (9) Å^3^	

### Data collection

**Table:**

Agilent SuperNova (Dual, Cu at zero) diffractometer with an Atlas detector	6103 independent reflections
Radiation source: fine-focus sealed tube	5505 reflections with *I* > 2σ(*I*)
Graphite monochromator	*R*_int_ = 0.019
Detector resolution: 10.4041 pixels mm^-1^	θ_max_ = 76.0°, θ_min_ = 2.9°
ω scans	*h* = −12→12
Absorption correction: multi-scan (*CrysAlis PRO*; Agilent, 2010)	*k* = −12→11
*T*_min_ = 0.548, *T*_max_ = 0.641	*l* = −19→17
11566 measured reflections	

### Refinement

**Table:**

Refinement on *F*^2^	Primary atom site location: structure-invariant direct methods
Least-squares matrix: full	Secondary atom site location: difference Fourier map
*R*[*F*^2^ > 2σ(*F*^2^)] = 0.069	Hydrogen site location: inferred from neighbouring sites
*wR*(*F*^2^) = 0.185	H-atom parameters constrained
*S* = 1.02	*w* = 1/[σ^2^(*F*_o_^2^) + (0.0942*P*)^2^ + 2.0717*P*] where *P* = (*F*_o_^2^ + 2*F*_c_^2^)/3
6103 reflections	(Δ/σ)_max_ < 0.001
381 parameters	Δρ_max_ = 0.94 e Å^−^^3^
0 restraints	Δρ_min_ = −0.46 e Å^−^^3^

### Special details

**Table:**

Geometry. All s.u.'s (except the s.u. in the dihedral angle between two l.s. planes) are estimated using the full covariance matrix. The cell s.u.'s are taken into account individually in the estimation of s.u.'s in distances, angles and torsion angles; correlations between s.u.'s in cell parameters are only used when they are defined by crystal symmetry. An approximate (isotropic) treatment of cell s.u.'s is used for estimating s.u.'s involving l.s. planes.
Refinement. Refinement of *F*^2^ against ALL reflections. The weighted *R*-factor *wR* and goodness of fit *S* are based on *F*^2^, conventional *R*-factors *R* are based on *F*, with *F* set to zero for negative *F*^2^. The threshold expression of *F*^2^ > σ(*F*^2^) is used only for calculating *R*-factors(gt) *etc*. and is not relevant to the choice of reflections for refinement. *R*-factors based on *F*^2^ are statistically about twice as large as those based on *F*, and *R*- factors based on ALL data will be even larger.

### Fractional atomic coordinates and isotropic or equivalent isotropic displacement parameters (Å^2^)

**Table:**

	*x*	*y*	*z*	*U*_iso_*/*U*_eq_	
S1	0.71402 (8)	0.06134 (7)	0.57164 (4)	0.0379 (2)	
S2	0.89812 (7)	0.71152 (6)	0.08641 (4)	0.03595 (19)	
O1	0.8589 (2)	0.0488 (2)	0.56504 (14)	0.0471 (5)	
O2	0.6524 (3)	0.0203 (2)	0.50369 (14)	0.0542 (6)	
O3	0.9112 (2)	0.8520 (2)	0.08884 (15)	0.0480 (5)	
O4	0.9752 (2)	0.6483 (2)	0.01369 (14)	0.0483 (5)	
N1	0.6393 (3)	−0.0306 (2)	0.66474 (16)	0.0382 (5)	
N2	0.9472 (3)	0.6333 (3)	0.17477 (16)	0.0404 (5)	
C1	0.6788 (4)	−0.0325 (3)	0.74573 (19)	0.0449 (7)	
C2	0.7995 (4)	0.0015 (3)	0.7654 (2)	0.0493 (7)	
H2	0.8760	0.0333	0.7202	0.059*	
C3	0.8076 (4)	−0.0119 (4)	0.8550 (2)	0.0553 (8)	
H3	0.8898	0.0140	0.8715	0.066*	
C4	0.6984 (4)	−0.0617 (3)	0.9187 (2)	0.0544 (9)	
H4	0.7090	−0.0756	0.9784	0.065*	
C5	0.5724 (4)	−0.0930 (3)	0.8997 (2)	0.0559 (9)	
H5	0.4975	−0.1239	0.9463	0.067*	
C6	0.5557 (4)	−0.0792 (3)	0.81181 (19)	0.0420 (7)	
C7	0.4485 (4)	−0.0954 (3)	0.7646 (2)	0.0482 (7)	
C8	0.4978 (4)	−0.0643 (3)	0.6777 (2)	0.0464 (7)	
H8	0.4464	−0.0648	0.6325	0.056*	
C9	0.3022 (4)	−0.1397 (3)	0.7985 (3)	0.0592 (9)	
H9	0.2490	−0.1537	0.7559	0.071*	
C10	0.2413 (5)	−0.1609 (4)	0.8818 (3)	0.0692 (11)	
H10A	0.2909	−0.1481	0.9265	0.083*	
H10B	0.1474	−0.1891	0.8979	0.083*	
C11	0.6616 (3)	0.2251 (3)	0.59101 (17)	0.0352 (6)	
C12	0.7527 (3)	0.3103 (3)	0.61593 (18)	0.0406 (6)	
H12	0.8434	0.2813	0.6204	0.049*	
C13	0.7080 (4)	0.4379 (3)	0.6340 (2)	0.0471 (7)	
H13	0.7696	0.4979	0.6499	0.057*	
C14	0.5751 (4)	0.4798 (3)	0.6294 (2)	0.0472 (7)	
C15	0.4850 (3)	0.3920 (3)	0.6042 (2)	0.0466 (7)	
H15	0.3938	0.4204	0.6006	0.056*	
C16	0.5281 (3)	0.2648 (3)	0.58467 (19)	0.0410 (6)	
H16	0.4676	0.2054	0.5672	0.049*	
C17	0.5264 (5)	0.6181 (3)	0.6508 (3)	0.0652 (11)	
H17A	0.5739	0.6469	0.6951	0.098*	
H17B	0.4263	0.6161	0.6745	0.098*	
H17C	0.5472	0.6811	0.5969	0.098*	
C18	0.9102 (3)	0.6694 (3)	0.2611 (2)	0.0421 (6)	
C19	0.8755 (3)	0.7930 (3)	0.2880 (2)	0.0485 (7)	
H19	0.8698	0.8696	0.2470	0.058*	
C20	0.8481 (4)	0.8009 (4)	0.3812 (2)	0.0560 (9)	
H20	0.8223	0.8846	0.4033	0.067*	
C21	0.8583 (4)	0.6908 (4)	0.4390 (2)	0.0584 (9)	
H21	0.8402	0.6998	0.5007	0.070*	
C22	0.8945 (4)	0.5654 (4)	0.4106 (2)	0.0507 (8)	
H22	0.9018	0.4896	0.4519	0.061*	
C23	0.9197 (3)	0.5538 (3)	0.32029 (18)	0.0365 (6)	
C24	0.9604 (3)	0.4422 (3)	0.2660 (2)	0.0427 (7)	
C25	0.9723 (3)	0.4917 (3)	0.18085 (19)	0.0414 (6)	
H25	0.9943	0.4404	0.1318	0.050*	
C26	0.9893 (3)	0.3026 (3)	0.2919 (2)	0.0493 (7)	
H26	1.0293	0.2505	0.2455	0.059*	
C27	0.9658 (4)	0.2395 (4)	0.3737 (2)	0.0627 (10)	
H27A	0.9260	0.2868	0.4227	0.075*	
H27B	0.9890	0.1473	0.3831	0.075*	
C28	0.7235 (3)	0.6688 (3)	0.10340 (16)	0.0350 (6)	
C29	0.6230 (3)	0.7598 (3)	0.13615 (18)	0.0406 (6)	
H29	0.6483	0.8447	0.1498	0.049*	
C30	0.4852 (3)	0.7244 (4)	0.14844 (19)	0.0475 (7)	
H30	0.4156	0.7863	0.1703	0.057*	
C31	0.4465 (4)	0.5999 (4)	0.1294 (2)	0.0528 (8)	
C32	0.5503 (4)	0.5089 (4)	0.0993 (2)	0.0510 (8)	
H32	0.5253	0.4224	0.0882	0.061*	
C33	0.6889 (3)	0.5426 (3)	0.08540 (19)	0.0428 (7)	
H33	0.7589	0.4807	0.0640	0.051*	
C34	0.2970 (4)	0.5637 (6)	0.1404 (3)	0.0743 (12)	
H34A	0.2841	0.4677	0.1603	0.111*	
H34B	0.2686	0.5838	0.0836	0.111*	
H34C	0.2404	0.6160	0.1845	0.111*	

### Atomic displacement parameters (Å^2^)

**Table:**

	*U*^11^	*U*^22^	*U*^33^	*U*^12^	*U*^13^	*U*^23^
S1	0.0481 (4)	0.0398 (4)	0.0250 (3)	0.0088 (3)	−0.0040 (3)	−0.0082 (2)
S2	0.0461 (4)	0.0315 (3)	0.0261 (3)	0.0029 (3)	0.0007 (3)	−0.0015 (2)
O1	0.0473 (12)	0.0503 (12)	0.0362 (11)	0.0102 (9)	0.0053 (9)	−0.0015 (9)
O2	0.0745 (16)	0.0575 (14)	0.0364 (11)	0.0124 (12)	−0.0174 (11)	−0.0212 (10)
O3	0.0620 (14)	0.0317 (10)	0.0446 (12)	−0.0025 (9)	0.0007 (10)	−0.0017 (8)
O4	0.0577 (13)	0.0453 (12)	0.0345 (11)	0.0038 (10)	0.0089 (9)	−0.0086 (9)
N1	0.0436 (13)	0.0332 (11)	0.0343 (12)	0.0008 (9)	0.0005 (10)	−0.0058 (9)
N2	0.0424 (13)	0.0452 (13)	0.0315 (12)	0.0022 (10)	−0.0054 (10)	−0.0004 (10)
C1	0.072 (2)	0.0307 (13)	0.0294 (14)	0.0035 (13)	−0.0069 (13)	−0.0019 (11)
C2	0.0564 (19)	0.0441 (16)	0.0438 (17)	0.0082 (14)	−0.0053 (14)	−0.0005 (13)
C3	0.067 (2)	0.0507 (19)	0.0508 (19)	0.0103 (16)	−0.0198 (17)	−0.0053 (15)
C4	0.081 (3)	0.0498 (18)	0.0329 (15)	0.0047 (17)	−0.0178 (16)	0.0056 (13)
C5	0.080 (3)	0.0447 (17)	0.0365 (16)	0.0042 (16)	−0.0017 (16)	0.0019 (13)
C6	0.0633 (19)	0.0284 (13)	0.0318 (14)	0.0089 (12)	−0.0043 (13)	−0.0045 (10)
C7	0.063 (2)	0.0314 (14)	0.0487 (17)	0.0036 (13)	−0.0063 (15)	−0.0068 (12)
C8	0.0548 (18)	0.0334 (14)	0.0509 (18)	0.0014 (13)	−0.0091 (14)	−0.0075 (12)
C9	0.055 (2)	0.0377 (16)	0.077 (3)	−0.0096 (14)	0.0078 (18)	−0.0125 (16)
C10	0.078 (3)	0.054 (2)	0.071 (3)	−0.0040 (19)	−0.003 (2)	−0.0106 (19)
C11	0.0450 (15)	0.0355 (13)	0.0231 (12)	0.0059 (11)	−0.0041 (10)	−0.0011 (10)
C12	0.0457 (16)	0.0435 (15)	0.0306 (13)	0.0015 (12)	−0.0054 (11)	−0.0006 (11)
C13	0.064 (2)	0.0373 (15)	0.0389 (15)	−0.0025 (14)	−0.0102 (14)	−0.0016 (12)
C14	0.068 (2)	0.0361 (15)	0.0346 (15)	0.0087 (14)	−0.0058 (14)	−0.0015 (11)
C15	0.0489 (17)	0.0452 (16)	0.0424 (16)	0.0120 (13)	−0.0062 (13)	0.0014 (13)
C16	0.0464 (16)	0.0416 (15)	0.0338 (14)	0.0045 (12)	−0.0078 (12)	−0.0009 (11)
C17	0.097 (3)	0.0376 (17)	0.058 (2)	0.0162 (18)	−0.011 (2)	−0.0059 (15)
C18	0.0384 (15)	0.0517 (17)	0.0356 (14)	−0.0015 (12)	−0.0042 (11)	−0.0082 (12)
C19	0.0467 (17)	0.0479 (17)	0.0505 (18)	−0.0021 (13)	−0.0081 (14)	−0.0068 (14)
C20	0.0501 (19)	0.059 (2)	0.059 (2)	−0.0021 (15)	−0.0004 (15)	−0.0293 (17)
C21	0.053 (2)	0.087 (3)	0.0372 (17)	−0.0017 (18)	−0.0054 (14)	−0.0227 (17)
C22	0.0513 (18)	0.064 (2)	0.0368 (16)	−0.0004 (15)	−0.0078 (13)	−0.0070 (14)
C23	0.0302 (13)	0.0460 (15)	0.0336 (13)	0.0001 (11)	−0.0072 (10)	−0.0049 (11)
C24	0.0399 (15)	0.0474 (17)	0.0406 (15)	0.0024 (12)	−0.0079 (12)	−0.0049 (12)
C25	0.0382 (15)	0.0496 (17)	0.0374 (15)	0.0048 (12)	−0.0064 (12)	−0.0136 (12)
C26	0.0469 (17)	0.0443 (17)	0.0538 (18)	0.0081 (13)	−0.0037 (14)	−0.0069 (14)
C27	0.075 (3)	0.061 (2)	0.0469 (19)	0.0113 (19)	−0.0055 (17)	0.0011 (16)
C28	0.0449 (15)	0.0355 (13)	0.0229 (11)	0.0054 (11)	−0.0057 (10)	0.0003 (10)
C29	0.0536 (17)	0.0387 (14)	0.0263 (13)	0.0097 (12)	−0.0037 (11)	−0.0006 (10)
C30	0.0484 (17)	0.062 (2)	0.0298 (14)	0.0139 (15)	−0.0043 (12)	−0.0055 (13)
C31	0.0482 (18)	0.079 (2)	0.0324 (15)	0.0026 (16)	−0.0108 (13)	−0.0078 (15)
C32	0.0564 (19)	0.0563 (19)	0.0450 (17)	−0.0009 (15)	−0.0182 (15)	−0.0112 (14)
C33	0.0543 (18)	0.0417 (15)	0.0350 (14)	0.0074 (13)	−0.0134 (12)	−0.0079 (12)
C34	0.050 (2)	0.121 (4)	0.057 (2)	−0.003 (2)	−0.0131 (17)	−0.028 (2)

### Geometric parameters (Å, º)

**Table:**

S1—O1	1.418 (2)	C15—C16	1.381 (4)
S1—O2	1.424 (2)	C15—H15	0.9500
S1—N1	1.664 (2)	C16—H16	0.9500
S1—C11	1.748 (3)	C17—H17A	0.9800
S2—O3	1.421 (2)	C17—H17B	0.9800
S2—O4	1.425 (2)	C17—H17C	0.9800
S2—N2	1.661 (2)	C18—C19	1.364 (5)
S2—C28	1.745 (3)	C18—C23	1.412 (4)
N1—C1	1.391 (4)	C19—C20	1.426 (5)
N1—C8	1.413 (4)	C19—H19	0.9500
N2—C18	1.398 (4)	C20—C21	1.360 (6)
N2—C25	1.431 (4)	C20—H20	0.9500
C1—C2	1.351 (5)	C21—C22	1.392 (5)
C1—C6	1.464 (4)	C21—H21	0.9500
C2—C3	1.403 (5)	C22—C23	1.389 (4)
C2—H2	0.9500	C22—H22	0.9500
C3—C4	1.363 (5)	C23—C24	1.473 (4)
C3—H3	0.9500	C24—C25	1.341 (4)
C4—C5	1.388 (6)	C24—C26	1.451 (4)
C4—H4	0.9500	C25—H25	0.9500
C5—C6	1.401 (4)	C26—C27	1.331 (5)
C5—H5	0.9500	C26—H26	0.9500
C6—C7	1.431 (5)	C27—H27A	0.9500
C7—C8	1.339 (5)	C27—H27B	0.9500
C7—C9	1.490 (5)	C28—C29	1.388 (4)
C8—H8	0.9500	C28—C33	1.390 (4)
C9—C10	1.304 (6)	C29—C30	1.383 (5)
C9—H9	0.9500	C29—H29	0.9500
C10—H10A	0.9500	C30—C31	1.392 (5)
C10—H10B	0.9500	C30—H30	0.9500
C11—C16	1.390 (4)	C31—C32	1.398 (5)
C11—C12	1.392 (4)	C31—C34	1.499 (5)
C12—C13	1.382 (4)	C32—C33	1.384 (5)
C12—H12	0.9500	C32—H32	0.9500
C13—C14	1.384 (5)	C33—H33	0.9500
C13—H13	0.9500	C34—H34A	0.9800
C14—C15	1.406 (5)	C34—H34B	0.9800
C14—C17	1.505 (4)	C34—H34C	0.9800
			
O1—S1—O2	119.94 (14)	C15—C16—C11	118.7 (3)
O1—S1—N1	106.60 (13)	C15—C16—H16	120.6
O2—S1—N1	106.14 (14)	C11—C16—H16	120.6
O1—S1—C11	110.00 (14)	C14—C17—H17A	109.5
O2—S1—C11	109.24 (14)	C14—C17—H17B	109.5
N1—S1—C11	103.58 (12)	H17A—C17—H17B	109.5
O3—S2—O4	120.11 (13)	C14—C17—H17C	109.5
O3—S2—N2	107.90 (14)	H17A—C17—H17C	109.5
O4—S2—N2	104.62 (13)	H17B—C17—H17C	109.5
O3—S2—C28	109.69 (14)	C19—C18—N2	128.6 (3)
O4—S2—C28	109.70 (14)	C19—C18—C23	123.3 (3)
N2—S2—C28	103.42 (12)	N2—C18—C23	108.0 (3)
C1—N1—C8	110.5 (3)	C18—C19—C20	116.2 (3)
C1—N1—S1	125.2 (2)	C18—C19—H19	121.9
C8—N1—S1	118.9 (2)	C20—C19—H19	121.9
C18—N2—C25	107.6 (2)	C21—C20—C19	121.2 (3)
C18—N2—S2	125.8 (2)	C21—C20—H20	119.4
C25—N2—S2	120.4 (2)	C19—C20—H20	119.4
C2—C1—N1	131.0 (3)	C20—C21—C22	121.9 (3)
C2—C1—C6	124.3 (3)	C20—C21—H21	119.0
N1—C1—C6	104.7 (3)	C22—C21—H21	119.0
C1—C2—C3	117.4 (3)	C23—C22—C21	118.4 (3)
C1—C2—H2	121.3	C23—C22—H22	120.8
C3—C2—H2	121.3	C21—C22—H22	120.8
C4—C3—C2	120.4 (4)	C22—C23—C18	119.0 (3)
C4—C3—H3	119.8	C22—C23—C24	134.2 (3)
C2—C3—H3	119.8	C18—C23—C24	106.8 (2)
C3—C4—C5	122.6 (3)	C25—C24—C26	122.2 (3)
C3—C4—H4	118.7	C25—C24—C23	107.5 (3)
C5—C4—H4	118.7	C26—C24—C23	130.3 (3)
C4—C5—C6	120.0 (3)	C24—C25—N2	110.0 (3)
C4—C5—H5	120.0	C24—C25—H25	125.0
C6—C5—H5	120.0	N2—C25—H25	125.0
C5—C6—C7	138.2 (3)	C27—C26—C24	127.1 (3)
C5—C6—C1	115.0 (3)	C27—C26—H26	116.4
C7—C6—C1	106.8 (3)	C24—C26—H26	116.4
C8—C7—C6	109.5 (3)	C26—C27—H27A	120.0
C8—C7—C9	120.7 (3)	C26—C27—H27B	120.0
C6—C7—C9	129.8 (3)	H27A—C27—H27B	120.0
C7—C8—N1	108.4 (3)	C29—C28—C33	121.6 (3)
C7—C8—H8	125.8	C29—C28—S2	119.6 (2)
N1—C8—H8	125.8	C33—C28—S2	118.8 (2)
C10—C9—C7	125.3 (4)	C30—C29—C28	118.7 (3)
C10—C9—H9	117.4	C30—C29—H29	120.7
C7—C9—H9	117.4	C28—C29—H29	120.7
C9—C10—H10A	120.0	C29—C30—C31	121.3 (3)
C9—C10—H10B	120.0	C29—C30—H30	119.4
H10A—C10—H10B	120.0	C31—C30—H30	119.4
C16—C11—C12	121.9 (3)	C30—C31—C32	118.5 (3)
C16—C11—S1	118.9 (2)	C30—C31—C34	121.0 (3)
C12—C11—S1	119.1 (2)	C32—C31—C34	120.4 (4)
C13—C12—C11	118.4 (3)	C33—C32—C31	121.2 (3)
C13—C12—H12	120.8	C33—C32—H32	119.4
C11—C12—H12	120.8	C31—C32—H32	119.4
C12—C13—C14	121.1 (3)	C32—C33—C28	118.6 (3)
C12—C13—H13	119.4	C32—C33—H33	120.7
C14—C13—H13	119.4	C28—C33—H33	120.7
C13—C14—C15	119.4 (3)	C31—C34—H34A	109.5
C13—C14—C17	120.8 (3)	C31—C34—H34B	109.5
C15—C14—C17	119.8 (3)	H34A—C34—H34B	109.5
C16—C15—C14	120.4 (3)	C31—C34—H34C	109.5
C16—C15—H15	119.8	H34A—C34—H34C	109.5
C14—C15—H15	119.8	H34B—C34—H34C	109.5
			
O1—S1—N1—C1	44.2 (3)	C12—C13—C14—C17	178.9 (3)
O2—S1—N1—C1	173.1 (2)	C13—C14—C15—C16	0.4 (5)
C11—S1—N1—C1	−71.9 (3)	C17—C14—C15—C16	−179.8 (3)
O1—S1—N1—C8	−164.4 (2)	C14—C15—C16—C11	0.4 (4)
O2—S1—N1—C8	−35.4 (2)	C12—C11—C16—C15	−0.4 (4)
C11—S1—N1—C8	79.6 (2)	S1—C11—C16—C15	176.9 (2)
O3—S2—N2—C18	44.1 (3)	C25—N2—C18—C19	−179.2 (3)
O4—S2—N2—C18	173.1 (2)	S2—N2—C18—C19	−27.2 (5)
C28—S2—N2—C18	−72.1 (3)	C25—N2—C18—C23	3.3 (3)
O3—S2—N2—C25	−167.1 (2)	S2—N2—C18—C23	155.4 (2)
O4—S2—N2—C25	−38.1 (3)	N2—C18—C19—C20	−177.3 (3)
C28—S2—N2—C25	76.7 (2)	C23—C18—C19—C20	−0.1 (5)
C8—N1—C1—C2	−174.9 (3)	C18—C19—C20—C21	0.8 (5)
S1—N1—C1—C2	−21.4 (5)	C19—C20—C21—C22	−0.6 (6)
C8—N1—C1—C6	3.9 (3)	C20—C21—C22—C23	−0.4 (5)
S1—N1—C1—C6	157.4 (2)	C21—C22—C23—C18	1.1 (5)
N1—C1—C2—C3	−179.9 (3)	C21—C22—C23—C24	179.4 (3)
C6—C1—C2—C3	1.5 (5)	C19—C18—C23—C22	−0.8 (5)
C1—C2—C3—C4	2.1 (5)	N2—C18—C23—C22	176.8 (3)
C2—C3—C4—C5	−4.3 (5)	C19—C18—C23—C24	−179.5 (3)
C3—C4—C5—C6	2.8 (5)	N2—C18—C23—C24	−1.9 (3)
C4—C5—C6—C7	−177.8 (3)	C22—C23—C24—C25	−178.7 (3)
C4—C5—C6—C1	0.7 (4)	C18—C23—C24—C25	−0.3 (3)
C2—C1—C6—C5	−2.9 (4)	C22—C23—C24—C26	−0.7 (6)
N1—C1—C6—C5	178.2 (3)	C18—C23—C24—C26	177.7 (3)
C2—C1—C6—C7	176.1 (3)	C26—C24—C25—N2	−175.9 (3)
N1—C1—C6—C7	−2.8 (3)	C23—C24—C25—N2	2.4 (3)
C5—C6—C7—C8	179.3 (4)	C18—N2—C25—C24	−3.6 (3)
C1—C6—C7—C8	0.7 (3)	S2—N2—C25—C24	−157.5 (2)
C5—C6—C7—C9	−1.0 (6)	C25—C24—C26—C27	−171.7 (4)
C1—C6—C7—C9	−179.6 (3)	C23—C24—C26—C27	10.5 (6)
C6—C7—C8—N1	1.7 (3)	O3—S2—C28—C29	−14.6 (3)
C9—C7—C8—N1	−178.0 (3)	O4—S2—C28—C29	−148.5 (2)
C1—N1—C8—C7	−3.7 (3)	N2—S2—C28—C29	100.3 (2)
S1—N1—C8—C7	−159.0 (2)	O3—S2—C28—C33	166.8 (2)
C8—C7—C9—C10	−173.7 (4)	O4—S2—C28—C33	32.8 (3)
C6—C7—C9—C10	6.6 (6)	N2—S2—C28—C33	−78.4 (2)
O1—S1—C11—C16	169.2 (2)	C33—C28—C29—C30	−1.9 (4)
O2—S1—C11—C16	35.6 (3)	S2—C28—C29—C30	179.5 (2)
N1—S1—C11—C16	−77.2 (2)	C28—C29—C30—C31	0.6 (4)
O1—S1—C11—C12	−13.5 (3)	C29—C30—C31—C32	1.5 (5)
O2—S1—C11—C12	−147.1 (2)	C29—C30—C31—C34	−178.2 (3)
N1—S1—C11—C12	100.1 (2)	C30—C31—C32—C33	−2.3 (5)
C16—C11—C12—C13	−0.5 (4)	C34—C31—C32—C33	177.4 (3)
S1—C11—C12—C13	−177.8 (2)	C31—C32—C33—C28	1.0 (5)
C11—C12—C13—C14	1.3 (4)	C29—C28—C33—C32	1.1 (4)
C12—C13—C14—C15	−1.3 (5)	S2—C28—C33—C32	179.8 (2)

### Hydrogen-bond geometry (Å, º)

Cg1, Cg2 and Cg3 are the centroids of the C1–C6, N2–C25 and C18–C23 rings, respectively.

**Table:**

*D*—H···*A*	*D*—H	H···*A*	*D*···*A*	*D*—H···*A*
C8—H8···O2^i^	0.95	2.50	3.433 (4)	166
C20—H20···O2^ii^	0.95	2.52	3.373 (5)	149
C25—H25···O4^iii^	0.95	2.48	3.406 (4)	166
C30—H30···*Cg*1^iv^	0.95	2.77	3.617 (4)	149
C34—H34*C*···*Cg*2^v^	0.98	2.95	3.525 (4)	119
C12—H12···*Cg*3^v^	0.95	2.87	3.739 (3)	153
C15—H15···*Cg*3^v^	0.95	2.86	3.638 (3)	140

Symmetry codes: (i) −*x*+1, −*y*, −*z*+1; (ii) *x*, *y*+1, *z*; (iii) −*x*+2, −*y*+1, −*z*; (iv) −*x*+1, −*y*+1, −*z*+1; (v) *x*−1, *y*, *z*.
